# ‘Autoreconstruction’ of the Mandible—Report of a Case

**DOI:** 10.3390/dj4020009

**Published:** 2016-04-13

**Authors:** Sarina E.C. Pichardo, Pieter de Roos, J.P. Richard van Merkesteyn

**Affiliations:** 1 Department of Oral & Maxillofacial Surgery (Chair: Prof. Dr. JPR van Merkesteyn), Leiden University Medical Center, P.O. Box 9600, 2300 RC LEIDEN, The Netherlands; s.e.c.pichardo@lumc.nl; 2 Department of Oral & Maxillofacial Surgery, IJsselland Hospital, Capelle aan den Ijssel; pdroos@vlietlandziekenhuis.nl

**Keywords:** bisphosphonates, osteonecrosis, jaws, osteomyelitis, neo-mandible

## Abstract

Bisphosphonate-related osteonecrosis of the jaw (BRONJ) was first mentioned in the literature in 2003. Since then, several reports have been published referring to this disease. The etiology of BRONJ still remains unclear. The treatment of BRONJ also remains a topic of discussion between those who are in favor of a conservative treatment and those who are convinced that surgical treatment gives the best results. In this case report, a patient is presented with BRONJ in the mandible which has been treated surgically in combination with antibiotic treatment. During surgery it appeared that a large part of the jaw was sequestrated full-thickness with, at the same time, formation of a substantial amount of subperiosteal bone that was formed around the BRONJ, supporting the sequestrated part of the mandible and, after sequestrectomy, serving as a neo-mandible. This case shows the capacity of the jawbone despite bisphosphonate use to regenerate itself.

## 1. Introduction

Bisphosphonate-related osteonecrosis of the jaw was first mentioned in the literature in 2003 [1]. Since then, several reports and research have been published referring to this disease. In the literature, authors are divided about the treatment. Some suggest to stay as conservative as possible, for surgical intervention could worsen the disease, leading to loss of (parts of) the jaw [2,3]. Other authors plead for a prompt surgical approach to stop the disease from extending in the jaw, thus preventing loss of continuity [4,5,6]. 

Subperiosteal bone is formed as a response to injury caused by inflammation, trauma to the bone, cancer or chronic irritation of the periosteum. It takes at least a few weeks before subperiosteal bone apposition is visible on an X-ray. Usually subperiosteal bone consists of a thin layer and is being resorbed in the normal bone turnover whenever the original stimulus has gone. Only in the relatively rare proliferative periostitis [7,8,9] or Garré’s osteomyelitis are larger quantities of subperiosteal bone found [10]. In older literature, however, cases of phosphorus necrosis of the jaw with abundant formation of subperiosteal bone are found. Thus, apart from the chronicity of the osteomyelitis seen in BRONJ, the use of bisphosphonates possibly plays a role in acquiring a large quantity of subperiosteal bone.

So far, it has never been seen or reported that BRONJ may lead to sequestration of a large part of the jaw with, at the same time, the presence of a substantial amount of subperiosteal bone that was formed around the BRONJ, supporting the sequestrated part of the mandible and, after sequestrectomy, serving as a neo-mandible. 

To our knowledge, this case report is the first in literature to report this phenomenon. 

## 2. Case Report

A 55-year-old woman with metastasized breast cancer for more than three years and multiple intraoral fistulas since six months prior was referred to the department of Oral and Maxillofacial Surgery of the Leiden University Medical Center. The medical history further showed deep vein thrombosis, appendectomy, hypercholesterolemia and hepatitis. The patient used Bactroban, Paracetamol/Codein, Zoladex, Innohep and Tamoxiphen. The patient also used Pamidronate for 27 months with a dose of 90 mg per month and Alendronate for 37 months orally with a dose of 70 mg per week. Before surgery, both anti-resorptive agents were stopped for one month, and after surgery they were not continued. The patient smoked 10–20 cigarettes a day, did not use alcohol, and stopped using drugs (marijuana, heroin) 32 years before. The patient did not receive radiotherapy in the head or neck region in the past.

**Figure 1:**
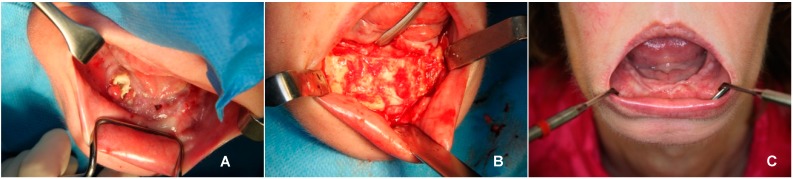
Photographs before, during and after surgery. (**A**) Multiple intraoral fistula and denuded bone; (**B**) Subperiosteal bone before closure of the surgical wound; (**C**) Intraoral view six weeks after surgery with closed mucosa.

At the presentation of pain, intraoral fistulas (Figure 1A) and an extraoral fistula in the submental region were found. Two months before, she had extractions of all her teeth under general anesthesia elsewhere because of caries and periodontitis, a productive submental fistula, and pain. Afterwards, she had an antimicrobial treatment for 10 days of Augmentin 625 (amoxicillin and clavulanic acid) and Perioaid mouth rinse. Despite the extractions, the pain and the fistulas persisted. 

**Figure 2:**
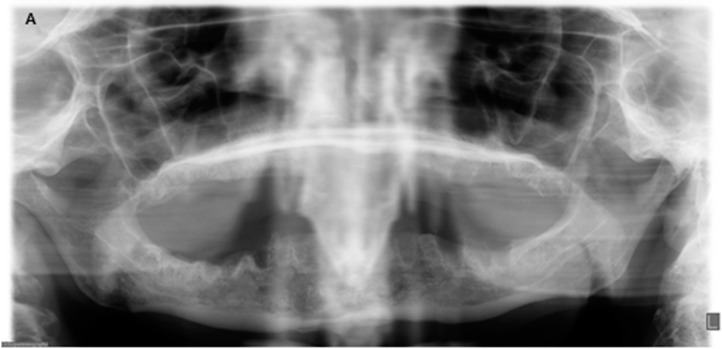

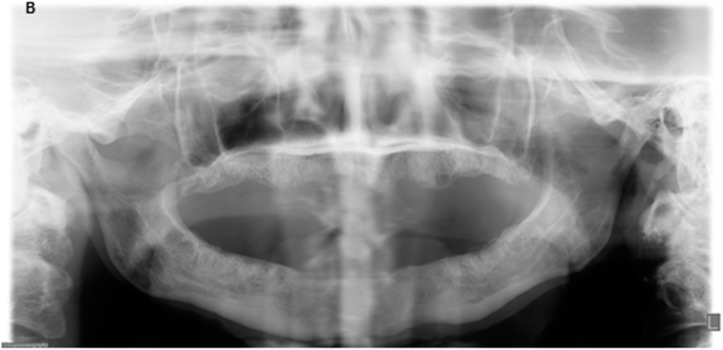
Radiologic findings before surgery and nine months after surgery. (**A**) Panoramic radiograph with extensive osteolysis, extending from region 46 to 34 up to the inferior border in the region of the symphyse; (**B**) Panoramic radiograph nine months post-operatively with healed, smooth edges of the mandibular body.

**Figure 3:**
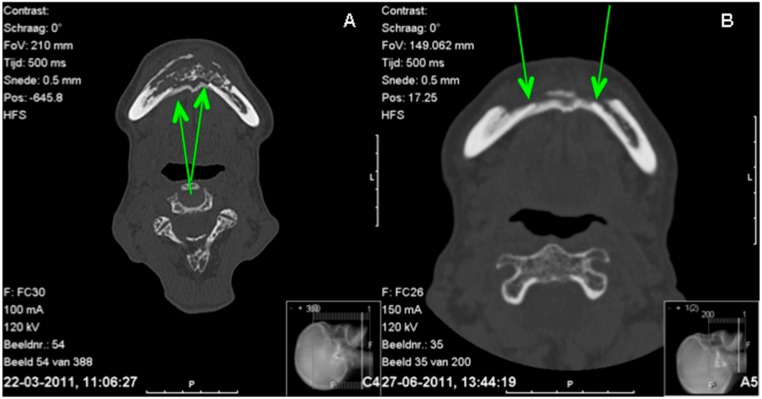
Comparison CT scans before (**A**) and three months after surgery (**B**). A = lingual subperiosteal bone can be seen and seems to connect both parts of the mandible; B = the difference between the cortex of the mandible and the subperiosteal bone is decreasing.

The panoramic radiograph showed osteolysis of the ventral part of the mandible (Figure 2). The CT scan (Figure 3A) showed massive osteolysis and sequestration of the ventral part of the mandible from region 34 to 45, matching osteomyelitis and BRONJ. The continuity of the mandible seemed intact just because of subperiostal bone formation (Figure 3A). 

A diagnosis of bisphosphonate-related osteonecrosis of the jaw was made. The patient was treated according to a protocol reported earlier by Alons [4] with a sequestrectomy under general anesthesia in combination with intravenous antibiotics. During surgery, the original mandible from region 34 to 45 appeared to be completely necrotized and sequestrated. The mental nerve could not be identified on the right side, and on the left side it could be identified. When the sequestrae were removed, a large quantity of subperiostal bone was found around the defect, especially at the former lingual border of the mandible. This subperiosteal bone seemed vital and perfused. After partial removal, its buccal shape was lowered and rounded off. Finally, the subperiosteal bone was shaped in order to make a primary closure without dead space possible and it seemed to have sufficient thickness to provide continuity of the mandible (Figure 1B). The wound was closed primarily in layers. The patient received an antimicrobial treatment according to protocol (Penicillin G (6 × 1 million EH) and Metronidazole (3 × 500 mg) were administered for five days intravenously, followed by Amoxicillin orally 3 × 500 mg for three weeks and Metronidazole 3 × 500 mg for three weeks). 

Histologic examination of the bone showed non-vital bone, signs of chronic inflammation and the extensive presence of microorganisms. *Streptococcus constellatus*, a mixed-cell infiltrate and *Actinomyces* were seen; there were no signs of metastases of the breast cancer in the mandible. 

The patient’s recovery was good without further complaints, intraoral dehiscences or fistulas (Figure 1C). During follow-up, no pathological fracture of the subperiosteal bone occurred. The panoramic radiograph showed continuity of the mandible and a cortex-like structure. The CT scan six weeks after surgery showed a lingual neo-cortex of the mandible without any signs of resorption (Figure 3B). At follow-up after nine months, the patient was still free of complaints.

## 3. Discussion

Bisphosphonates are built in bone tissue and are released after cessation of therapy over a prolonged time. Therefore, bisphosphonates stay effective for years. Since bisphosphonates inhibit the osteoclasts, bone resorption is decreased, including, in this case, probably also the subperiosteal bone resorption. 

The reason the subperiosteal bone grew to this volume is probably because of the long duration of chronic irritation of the periosteum caused by the former dentition with multiple inflammatory foci and the long-term use of bisphosphonates. However, subperiosteal bone is supposed to be resorbed entirely in the normal bone remodeling process. However, in this case, it did not. A possible explanation for this could be due to the bisphosphonates, which decrease (subperiosteal) bone resorption. In normal patients, this amount of subperiosteal bone formation would not have been reached due to the normal bone remodeling process and normal (subperiosteal) bone resorption. In our opinion, there is not necessarily more subperiosteal bone formation in BRONJ patients compared to normal patients, but rather a decreased bone resorption due to bisphosphonates. 

The pre- and post-operative CT scan confirmed this finding: that the continuity of the original lingual cortex of the region from 34 to 45 was gone and replaced by subperiosteal bone (Figure 3B). 

The CT scan also showed that the subperiostal bone developed a cortex-like structure (Figure 3B). The distinction between the former cortex of the mandible and the cortex of the neo-mandible was visible on the CT scan (Figure 3B). Where the first CT scan made at presentation clearly shows a distinction between the subperiosteal bone and the lingual cortex, the second CT scan made several weeks after presentation appears to have no such clear distinction anymore. It seems as if a new cortex has been formed. 

It appears that this phenomenon is not entirely new. Older literature going back to the mid-19th century already showed subperiosteal bone formation in phossy jaw patients during and after surgery [11,12]. Workers in the match industry were at risk for developing phossy jaw caused by the inhalation of phosphorus vapors in the factories. These phosphorus vapors had a similar effect on the jawbone as bisphosphonates do [13,14,15,16]. Several written case reports of phossy jaw patients are comparable in clinical features with the current BRONJ, including, in several cases, abundant subperiosteal bone formation [11,12,17]. In this case, the subperiosteal bone mass appeared sufficient to retain mandibular continuity during a follow up of more than nine months. 

## 4. Conclusions

This report of a case of BRONJ of the mandible with excessive subperiosteal bone formation shows a practical and patient-friendly use of the excessive amount of this subperiosteal bone in BRONJ.
